# Supplementary material for: Fertilizers and Fertilization Strategies Mitigating Soil Factors Constraining Efficiency of Nitrogen in Plant Production
Source: Plants (Basel). 2022 Jul 15;11(14):1855. doi: 10.3390/plants11141855 (PMC9319167; doi:10.3390/plants11141855)
Supplement: Supplementary file 1 [file plants-11-01855-s001.zip › plants-1816768-supplementary.pdf]

Supplementary material

Fertilizers as means of production mitigating soil factors constraining efficiency of nitrogen in plant production

Przemysław Barłóg[1]*, Witold Grzebisz[1] and Remigiusz Łukowiak[1]

Department of Agricultural Chemistry and Environmental Biogeochemistry, Poznan University of Life Sciences, Wojska Polskiego 71F, 60-625 Poznan, Poland; witold.grzebisz@up.poznan.pl (W.G.); remigiusz.lukowiak@up.poznan.pl (R.Ł.)
* Correspondence: przemyslaw.barlog@up.poznan.pl; Tel.: +48-618-48-77-88 (P.B.)

Table S1. A detailed analysis and evaluation of agronomic factors responsible for nitrogen gap (NG)*

**Field characteristics**	**Field number → decreasing Nitrogen Gap**			
	**13**	**10**	**5**	**14**
N_f rate, kg ha^{-1}	150	160	130	130
Nitrogen gap/gain, kg N ha^{-1}	−59	−40	−14	+4
Yield gap/gain, kg ha^{-1}	−3729	−2664	−858	+242
Soil usability class	Very low	Low	Low	Low
Fore-crop	Low	Medium	Medium	Medium
Variety	Extensive	Extensive	Extensive	Extensive
Sowing term	Adequate	Adequate	Adequate	Adequate
Manure	Lack	Lack	Lack	Lack
Soil reaction (pH)	Acidic	Slightly acid	Slightly acid	Slightly acid
Phosphorus content – class	Medium	Medium	Medium	Medium
Potassium content – class	Low	High	Medium	High
Magnesium content class	Medium	Medium	Low	Medium
Fungicide protection	Medium	Medium	Medium	Medium

Key: Low, Medium, High, Adequate, – a relative range of the growth factor.; Acidic, slightly acid, neutral – ranges of soil pH.

*The calculation NG procedure consists of a set of formulas:

- Partial Factor Productivity of N_f: $PFP_{Nf} = \frac{Ya}{N_f}$ $(kg\ kg^{-1}\ N_f)$
- Attainable, maximum yield: $Y_{attmax} = cPFP_{Nf} \times N_f$ $(t\ or\ kg\ ha^{-1})$
- Yield Gap: $YG = Y_{attmax} - Y_a$ $(t\ ha^{-1})$
- Nitrogen Gap: $NG = \frac{YG}{cPFP_{Nf}}$ $(kg\ N\ ha^{-1})$

where: PFP_{Nf} – partial factor productivity of N_f, kg grain/seeds, tubers etc. per kg N_f; Y_a – actual yield of a currently grown crop, t ha^{-1}; N_f – the amount of applied fertilizer N, kg ha^{-1}; Y_{attmax} – the maximum attainable yield, t ha^{-1}; $cPFP_{Nf}$ – the average of the third quartile (Q3) of the set of PFP_{Nf} indices arranged in ascending order, kg grain/seeds, tubers etc. per kg N_f; YG – yield gap, t ha^{-1}; NG – nitrogen gap, kg ha^{-1} of N.
